# Supplementary material for: The Role of Chemotherapy in Patients with Synchronous Colorectal Liver Metastases: A Nationwide Study
Source: Cancers (Basel). 2025 Mar 13;17(6):970. doi: 10.3390/cancers17060970 (PMC11940559; doi:10.3390/cancers17060970)
Supplement: Supplementary file 1 [file cancers-17-00970-s001.zip › cancers-3445391-supplementary.pdf]

Table S1. Multivariable analysis of patients who were treated with adjuvant chemotherapy.

	Univariable HR, 95% CI	P value	Multivariable HR, 95% CI	P value
**Age (years)**				
<70	Reference		Reference	
≥70	(1.17-1.71)	<0.001	1.42 (1.17-1.71)	<0.001
**Gender**				
Women	Reference		Reference	
Men	(0.78-1.25)	0.928	1.02 (0.85-1.22)	0.850
ASA				
1-2	Reference		Reference	
3-4	(0.95-1.86)	0.094	1.02 (0.82-1.28)	0.847
**T category of primary cancer**				
T1-T2	Reference		Reference	
T3-T4	(1.87-7.73)	<0.001	1.24 (0.89-1.72)	0.202
**Lymphatic spread of primary cancer**				
N0	Reference		Reference	
N1-N2	(1.33-2.83)	<0.001	1.74 (1.40-2.17)	<0.001
**Chemotherapy**				
Upfront surgery	Reference		Reference	
Neoadjuvant	0.75 (0.63-0.90)		1.04 (0.81-1.34)	0.743
**Number of liver metastases**				
1	Reference		Reference	
2	1.37 (1.09-1.71)	0.007	1.37 (1.08-1.74)	0.010
3-5	1.48 (1.21-1.82)	<0.001	1.34 (1.07-1.68)	0.010
6	1.80 (1.39-2.33)	<0.001	1.57 (1.18-2.01)	0.002
>6	3.36 (2.19-5.15)	<0.001	3.58 (2.16-5.94)	<0.001

Table S2. Multivariable analysis of patients who were treated with neoadjuvant chemotherapy.

	Univariable HR, 95% CI	P value	Multivariable HR, 95% CI	P value
**Age (years)**				
<70	Reference		Reference	
≥70	1.38 (1.19-1.61)	<0.001	1.47 (1.23-1.75)	0.004
**Gender**				
Women	Reference		Reference	
Men	0.92 (0.80-1.07)	0.287	0.96 (0.81-1.14)	0.632
ASA				
1-2	Reference		Reference	
3-4	1.20 (1.01-1.42)	0.041	1.12 (0.92-1.37)	0.266
**T category of primary cancer**				
T1-T2	Reference		Reference	
T3-T4	1.81 (1.40-2.37)	<0.001	1.40 (1.04-1.85)	0.025
**Lymphatic spread of primary cancer**				
N0	Reference		Reference	
N1-N2	1.86 (1.55-2.24)	<0.001	1.67 (1.37-2.05)	<0.001
**Chemotherapy**				
No Adjuvant chemotherapy	Reference		Reference	
Adjuvant chemotherapy	0.77 (0.65-0.90)	0.001	0.82 (0.68-0.97)	0.035
**Number of liver metastases**				
1	Reference		Reference	
2	1.24 (1.00-1.53)	0.048	1.41 (1.11-1.78)	0.004
3-5	1.24 (1.02-1.51)	0.028	1.33 (1.01-1.66)	0.009
6	1.51 (1.20-1.89)	<0.001	1.49 (1.15-1.93)	0.003
>6	2.50 (1.81-3.46)	<0.001	2.26 (1.48-3.45)	<0.001
